# Supplementary material for: Conditional chemoconnectomics (cCCTomics) as a strategy for efficient and conditional targeting of chemical transmission
Source: eLife. 2024 Apr 30;12:RP91927. doi: 10.7554/eLife.91927 (PMC11060718; doi:10.7554/eLife.91927)
Supplement: Figure 3—source data 3. [file elife-91927-fig3-data3.zip › Figure 3 source data3 part 2.zip!/Figure 3-Source data 3-part2/1063-1-CRISPResso_Report_az97gqtx.zip!/CRISPResso_on_1063_1/4a.Combined_insertion_deletion_substitution_locations.pdf]

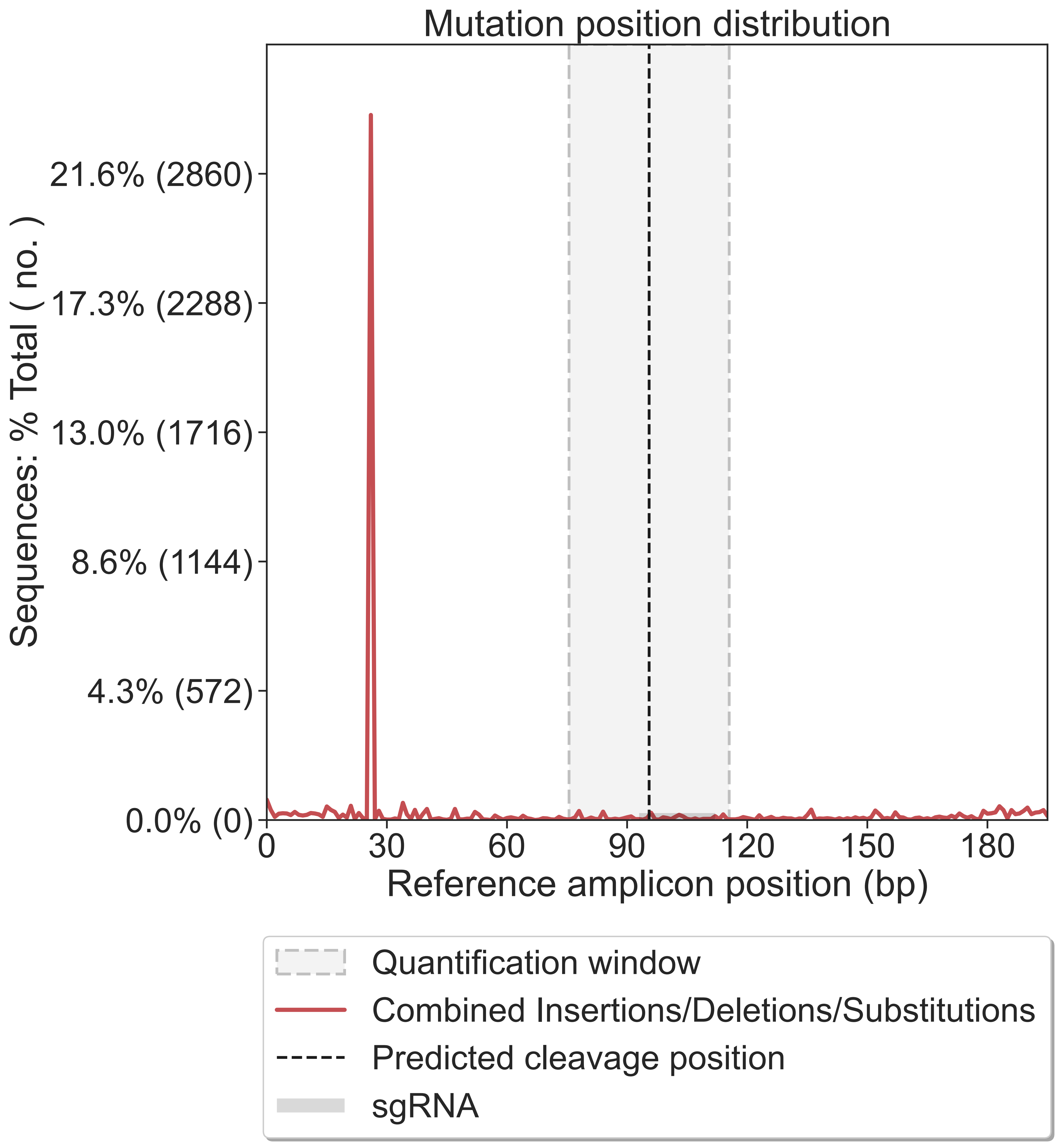
Mutation position distribution
21.6% (2860)
17.3% (2288)
13.0% (1716)
8.6% (1144)
4.3% (572)
0.0% (0)
Sequences: % Total (no.)
0
30
60
90
120
150
180
Reference amplicon position (bp)
Quantification window
Combined Insertions/Deletions/Substitutions
Predicted cleavage position
sgRNA
